# Open(G)PIAS: An Open-Source Solution for the Construction of a High-Precision Acoustic Startle Response Setup for Tinnitus Screening and Threshold Estimation in Rodents

**DOI:** 10.3389/fnbeh.2019.00140

**Published:** 2019-06-25

**Authors:** Richard Gerum, Hinrich Rahlfs, Matthias Streb, Patrick Krauss, Jannik Grimm, Claus Metzner, Konstantin Tziridis, Michael Günther, Holger Schulze, Walter Kellermann, Achim Schilling

**Affiliations:** ^1^Biophysics Group, Department of Physics, Center for Medical Physics and Technology, Friedrich-Alexander University Erlangen-Nürnberg (FAU), Erlangen, Germany; ^2^Experimental Otolaryngology, ENT-Hospital, Head and Neck Surgery, Friedrich-Alexander University Erlangen-Nürnberg (FAU), Erlangen, Germany; ^3^Multimedia Communications and Signal Processing, Friedrich-Alexander University Erlangen-Nürnberg (FAU), Erlangen, Germany; ^4^Cognitive Computational Neuroscience Group at the Chair of English Philology and Linguistics, Department of English and American Studies, Friedrich-Alexander University Erlangen-Nürnberg (FAU), Erlangen, Germany

**Keywords:** low-cost setup, Anaconda package, tinnitus, animal model, startle, 3D acceleration sensor

## Abstract

The modulation of the acoustic startle reflex (ASR) by a pre-stimulus called pre-pulse inhibition (PPI, for gap of silence pre-stimulus: GPIAS) is a versatile tool to, e.g., estimate hearing thresholds or identify subjective tinnitus percepts in rodents. A proper application of these paradigms depends on a reliable measurement of the ASR amplitudes and an exact stimulus presentation in terms of frequency and intensity. Here, we introduce a novel open-source solution for the construction of a low-cost ASR setup. The complete software for data acquisition and stimulus presentation is written in Python 3.6 and is provided as an Anaconda package. Furthermore, we provide a construction plan for the sensor system based on low-cost hardware components. Exemplary GPIAS data from two animal models (*Mus musculus, Meriones unguiculatus*) show that the ratio histograms (1-GPIAS) of the gap-pre-stimulus and no pre-stimulus ASR amplitudes can be well described by a log-normal distribution being in good accordance to previous studies with already established setups. Furthermore, it can be shown that the PPI as a function of pre-stimulus intensity (threshold paradigm) can be approximated with a hard-sigmoid function enabling a reproducible sensory threshold estimation. Thus, we show that the open-source solution could help to further establish the ASR method in many laboratories and, thus, facilitate and standardize research in animal models of tinnitus and/or hearing loss.

## Introduction

A behavioral paradigm that can be used to assess hearing abilities in animal models (i.e., behavioral audiometry) without the necessity to apply time-consuming conditioning paradigms is the so called pre-pulse inhibition of the acoustic startle reflex (PPI of ASR).

The startle reflex is induced by an intense stimulus such as a loud tone. This reflex, processed in the brainstem, is an evolutionary adaptation to prevent subjects from harm, e.g., by sudden attacks of predators (Koch, 1999). The reaction to a loud tone, the acoustic startle reflex (ASR), can be modulated by a variety of different factors such as drug treatment, pathological conditions, or presentation of pre-stimuli (Koch, 1999). A decrease of the ASR amplitude caused by any kind of pre-stimulus is called pre-pulse inhibition (PPI) and indicates that the stimulus has actually been perceived (Fendt et al., 2001). Hence, for example, hearing thresholds can be determined by acoustic pre-stimuli such as pure tones of varying intensity (Tziridis et al., 2012; Walter et al., 2012).

Furthermore, Turner et al. (2006) suggested a novel paradigm using the ASR as a tool for tinnitus screening. The paradigm is based on the fact that not only a tone or noise pre-stimulus can reduce the startle response but also a gap of silence embedded in band pass filtered noise can lead to a suppression of the ASR. This paradigm is called “Gap-Pre-Pulse Inhibition of the Acoustic Startle Reflex” (GPIAS). Thus, a potential tinnitus percept leads to a masking of this gap of silence and consequently results in a diminished decrease of the startle amplitudes (decrease of GPIAS) (Turner et al., 2006). It is advantageous that this paradigm does not depend on any pre-training of the animals such as classical conditioning (e.g., Jastreboff et al., 1988), because in neurophysiological studies it is difficult to separate cortical plasticity induced by tinnitus development from plasticity induced from learning. In addition, this paradigm is less time consuming compared to conditioning paradigms. For these reasons, this paradigm is widely used in the tinnitus research community (Kalappa et al., 2014; Krauss et al., 2016; Shore et al., 2016; Pienkowski, 2017). Due to its wide acceptance, the GPIAS paradigm and the evaluation procedures are continuously advanced and improved (Longenecker and Galazyuk, 2012; Schilling et al., 2017). Nevertheless, the validity of the paradigm including the “filling in hypothesis are still under debate” (cf. Eggermont, 2013). Thus, further effort needs to be invested in the validation process of the GPIAS paradigm.

However, setups for the recording of ASR responses for tinnitus screening as well as hearing ability estimation are still quite expensive and cannot be adjusted to the question at hand. Our aim is to provide an open-source solution for the construction of an ASR setup that is written in the interpreted programming language Python and is based on commercially available hardware components. We demonstrate the efficacy of our setup by exemplary results from our animal models (mouse: *Mus musculus* and Mongolian gerbil: *Meriones unguiculatus*).

## Materials and Methods

###  Animals and Housing

The Mongolian gerbils were housed in standard animal racks (Bio A.S. Vent Light, Ehret Labor- und Pharmatechnik, Emmendingen, Germany) in groups of 3–4 animals with free access to water and food at a room temperature of 20–25°C under a 12/12 h dark/light circle. The mice (type: C57/BL/6 “J”Crl, age: ≈10 weeks) were housed in groups of two animals under equal conditions. The care of the animals was approved by the state of Bavaria (Regierungspräsidium Mittelfranken, Ansbach, Germany, No. 54-2532.1-02/13 and Regierungspräsidium, Würzburg, Germany, No. 55.2-2532-2-137). Exemplary measurements were recorded using 8 male Mongolian gerbils purchased from the Leibniz Institute for Neurobiology in Magdeburg and 10 male mice purchased from Charles River Laboratories.

###  Software

The complete software for the ASR setup is written in Python 3.6 (Van Rossum and Drake, 1995), an interpreted programming language optimized for scientific purposes. For maximum efficiency several open-source libraries are used. For numerical operations, such as matrix operations, the Numpy library is used (van der Walt et al., 2011). Further complex mathematical operations, such as signal filter functions, are implemented using the SciPy package (Olifant, 2007). The data are visualized using the Matplotlib library (Hunter, 2007) and the Pylustrator (Gerum, 2018) and stored using the Pandas library (McKinney, 2010).

## Setup

**Figure 1:**
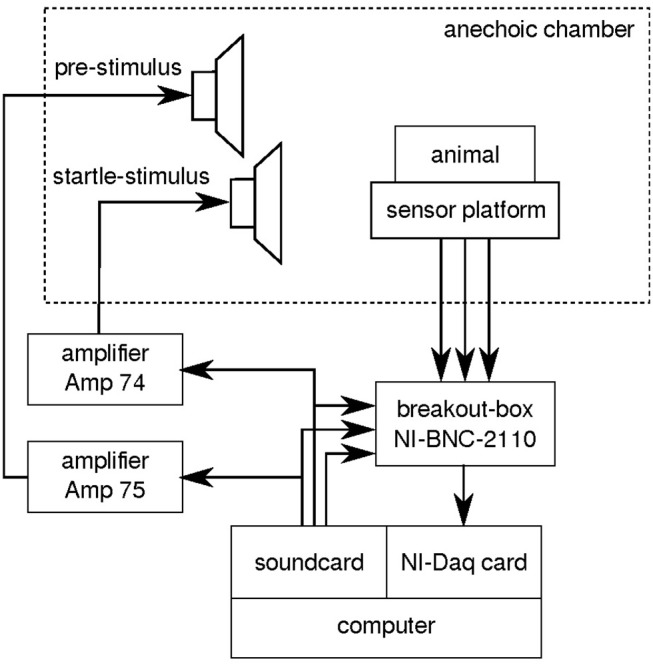
ASR measurement setup. Stimuli are presented via two different loudspeakers, one for the pre-stimulus and one for the startle stimulus, both placed in an anechoic chamber. Both loudspeakers are connected via an amplifier (Amp 74 and 75) to the soundcard of the computer. The animal is restrained in an acrylic tube, located on a sensor platform in the anechoic chamber. ASR amplitudes are measured via an acceleration sensor. Sound and accelerations are recorded using a data acquisition card connected to the PC over a breakout box. Our open-source ASR program (Python) controls stimulus application and measurement recording.

The setup is located in an anechoic chamber (Industrial Acoustics Company GmbH, Niederkrüchten, Germany) and consists of two main parts: the stimulation hardware and the recording hardware (see Figure 1). The animal is restrained in an acrylic tube (different inner diameter: 27, 37, or 42 mm, depending on the size of the animal) placed on a sensor platform with an integrated acceleration sensor (ADXL 335 on GY 61 board, Robotpark). All calibration measurements were made in the restrainer to correct for acoustical perturbations. However, it could be useful to develop a more acoustically transparent material (Longenecker and Galazyuk, 2012). The animal is acoustically stimulated using two different loudspeakers. A broadband two-way loudspeaker (Canton Plus XS.2) presents the pre-stimuli such as narrowband noise or pure tones of low amplitudes. To protect the broadband loudspeaker from damage by loud stimuli, a second loudspeaker (Neo-25s, Sinuslive) is used to present the startle stimuli. The startle stimuli causing the animal to twitch (ASR-amplitudes) are 20 ms noise bursts, with an intensity of 115 dB SPL, proven to be a valid stimulus to induce an ASR response (cf. Turner et al., 2006). Note that for different species it can be helpful to choose a different startle stimulus to guarantee stable startle responses (Longenecker and Galazyuk, 2012). In this study the same startle stimulus is used for all paradigms (e.g., threshold or GPIAS measurements). All stimuli are presented via a soundcard (Asus Xonar STX II, used sampling rate: 96 kHz) connected to two pre-amplifiers (Amp 75 for pre-stimuli, Amp 74 for startle stimuli, Thomas Wulf, Frankfurt).

The recording and digitization of the analog signal of the acceleration sensor (ADXL 335 on GY 61 board, Robotpark) is performed by a data acquisition card (PCIe-6320, National Instruments) connected to the sensor via a breakout box (NI-BNC 2110). The synchronization of startle stimulus onset and ASR response is assured by a trigger pulse generated by the soundcard and sent directly to the data acquisition card. The rising edge of the trigger pulse is used to align the measured ASR response to the startle stimulus onset.

**Figure 2:**
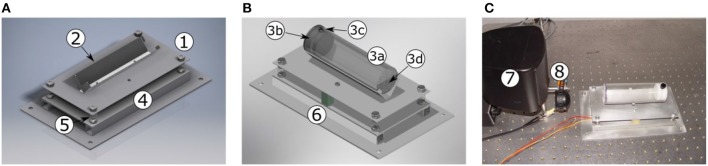
Sensor platform for ASR quantification. **(A)** Sensor platform (without animal restrainer). **(B)** Sensor platform (with animal restrainer). **(C)** Photo of the sensor platform in front to the loudspeakers. The upper plate (1) of the sensor platform holds the mount (2) for the animal restrainer (3). The restrainer consists of an acrylic tube (3a) fixed in the mount, closed by a 3D printed plastic cap (3b), fixed with a screw (3c), at the rear end, and a wire-mesh (3d) at the front end, facing the loudspeaker. The acceleration sensor is fixed underneath the upper plate. This plate is flexibly mounted on the lower plate (4) via four springs (5). The lower plate is screwed to the vibration-isolated table. Rubber foam (6) prevents the sensor system from oscillating. The sensor platform is placed in front of the two speakers: pre-stimulus (7) and startle stimulus loudspeaker (8).

The sensor platform consists of two plates (cf. Figure 2A1,4). The lower plate is fixed to the vibration-isolated table (TMC, Peabody, MA, USA) by four screws. The upper plate is flexibly mounted on the lower plate by four springs, damped by two foam rubber blocks. The upper plate has a mount for the animal restrainer. This restrainer consists of an acrylic tube which is closed on both ends, with (Figure 2B) a wire mesh at the front end, facing the speakers, and a plastic cap (custom 3D print) at the rear end. The wire mesh causes no measurable distortions in the spectra of the acoustic stimuli and the cap prevents the animal from escaping the restrainer. The fixed mount ensures a constant distance between the animal and the loudspeakers (Figure 2C).

As described above, ASR amplitudes are quantified by a three-way acceleration sensor, fixed underneath the upper plate of the sensor platform. When the animal twitches, as a response to the startle stimulus, the upper plate moves. This movement is quantified by the acceleration sensor. The ASR amplitude is

(1) $$\begin{array}{r} A={\text{max}}_{t}\left(a\left(t\right)\cdot \Theta \left(t\right)\cdot \Theta \left(150\text{ms}-t\right)\right), \end{array}$$

where Θ is the Heaviside function, *t* = 0 the begin of the startle stimulus and

(2) $$\begin{array}{r} a\left(t\right)=\sqrt{{\left(c_{x}\cdot a_{x\left(t\right)}\right)}^{2}+{\left(c_{y}\cdot a_{y\left(t\right)}\right)}^{2}+{\left(c_{z}\cdot a_{z\left(t\right)}\right)}^{2}}, \end{array}$$

with *a*_*x*(*t*)_, *a*_*y*(*t*)_, *a*_*z*(*t*)_, the measured acceleration in *x, y, z* direction and *c*_*x*_, *c*_*y*_, *c*_*z*_ the calibrated factors, so that same force leads to same acceleration.

## Stimulation Software

The software consists of several parts: a configuration module that allows to adapt the software to the given hardware setting, a protocol generator that allows to prepare the stimuli that should be presented, and a measurement module that applies the specified stimuli to the animal and records the responses.

### Configuration

The configuration module allows to specify details of the used hardware: The soundcard and sound driver as well as the channels for trigger pulse, pre-stimulus, and startle stimulus can be specified.

The configuration module also allows to perform some calibration measurements, e.g., for synchronizing the output channels of the soundcard or equalizing the frequency response of the loudspeakers.

To calibrate possible latency shifts, a TTL pulse is presented in all selected output channels and the results are recorded using the data acquisition card. The program uses these measurements to determine the relative time shifts between the channels.

**Figure 3:**
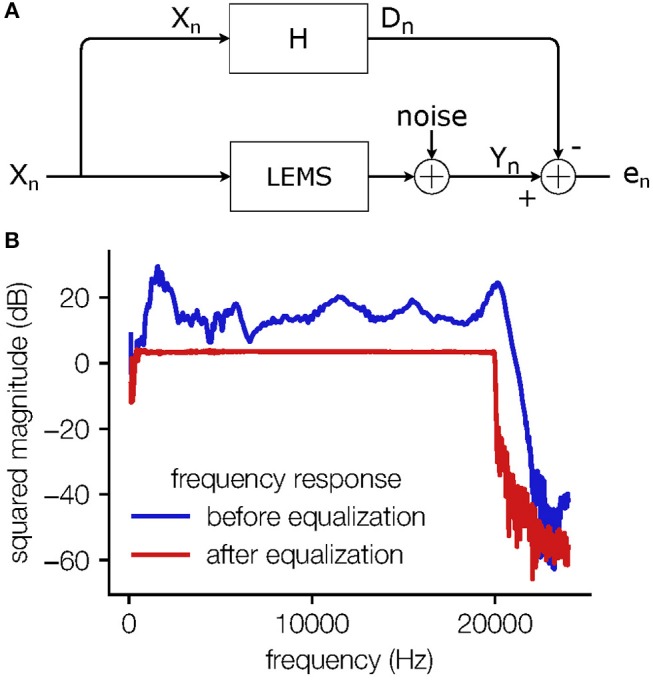
Equalizer scheme. **(A)** The block diagram of the system identification task; the output signal *X*_*n*_ of the soundcard is fed into the Loudspeaker-Enclosure-Microphone-System (LEMS) and recorded by the microphone. The measurement noise is modeled here separately as being added in the discrete time domain. The same signal *X*_*n*_ is fed into the adaptive filter *H* and the difference signal *e*_*n*_ is used to adjust the filter coefficients. This iterative process leads to a filter that mimics the transfer function of the “unknown system”. **(B)** The frequency response of the overall system without equalization (blue) and with equalization (red). The frequency response is flattened in the range from 2 to 20 kHz.

Since loudspeakers often exhibit a non-flat frequency response, it is desirable to correct these deviations using an equalizing filter. To measure the frequency response of the loudspeaker, a microphone [condenser microphone (B and K Type 4190), pre-amplifier (B and K Type 2669), measuring amplifier (B and K Type 2610)] is placed at the position of the animal in the measurement setup and the influence of the animal itself on the sound field is emulated by a piece of rubber foam. The coefficients of the equalizer filter are determined by first identifying the loudspeaker-enclosure-microphone system (LEMS) and subsequently inverting the minimum-phase component of its transfer function in the frequency domain. The system identification task, depicted schematically in Figure 3A, is solved by an adaptive linear filter using the NLMS algorithm (Widrow et al., 1976), which iteratively minimizes the power of the error signal between the adaptive filter output *D*_*n*_ and the observed microphone signal *Y*_*n*_ for a known excitation signal *X*_*n*_. To simplify the identification of all frequencies of interest, the excitation signal should exhibit a constant power spectral density in the relevant frequency range as provided, e.g., by a white noise sequence or pseudo-random maximum-length sequences (Rife and Vanderkooy, 1989). Finally, the time-domain coefficients of the equalizer filter are obtained by frequency bin-wise inversion of the identified transfer function over the desired frequency range and subsequent application of the inverse discrete Fourier transform (IDFT), realized by an Inverse Fast Fourier Transform (IFFT). By convolving the loudspeaker driving signals with the equalization filter before playback, a flat frequency response for the overall system, i.e., the cascade of the equalization filter and the loudspeaker-enclosure-microphone system can be achieved as depicted in Figure 3B.

### Protocols

The protocol module allows to define measurement protocols for different paradigms. Currently two types of protocols are implemented: hearing threshold measurements (based on PPI of ASR) and GPIAS measurements, but the system is flexible to allow the future implementation of additional protocols.

Typical measurement sessions consist of multiple trials, each ending with a startle stimulus. At the beginning of each measurement session, five trials without pre-stimulus are presented to prevent adaptation effects in the response amplitudes of the animal during the subsequent trials. It could potentially be beneficial to perform some acclimatization sessions to reduce stress and thus to get more reliable results (Longenecker et al., 2018).

For hearing threshold determination, some trials have a pre-stimulus in the form of a short (40 ms) pure tone 100 ms before the startle stimulus. For the protocol, a frequency range (with octave, 1/2 octave, or 1/4 octave steps), a sound pressure level range, and a trial repetition count can be specified. The number of trials with pre-stimulus is the same as the number of trials without a pre-stimulus.

For the GPIAS paradigm, each trial has a noise presented before the startle stimulus. The noise can be broadband or bandlimited around a center frequency. For each measurement different center frequencies can be specified, as well as the number of measurement repetitions. The noise can be interrupted by a short (50 ms, smoothed with 20 ms sin^2^-ramps) gap of silence. The number of trials with a gap is the same as the number of trials without a gap.

The trials in each protocol are pseudo randomized to prevent habituation effects of the animal. The same pseudo random order is used for all animals.

### Measurement

**Figure 4:**
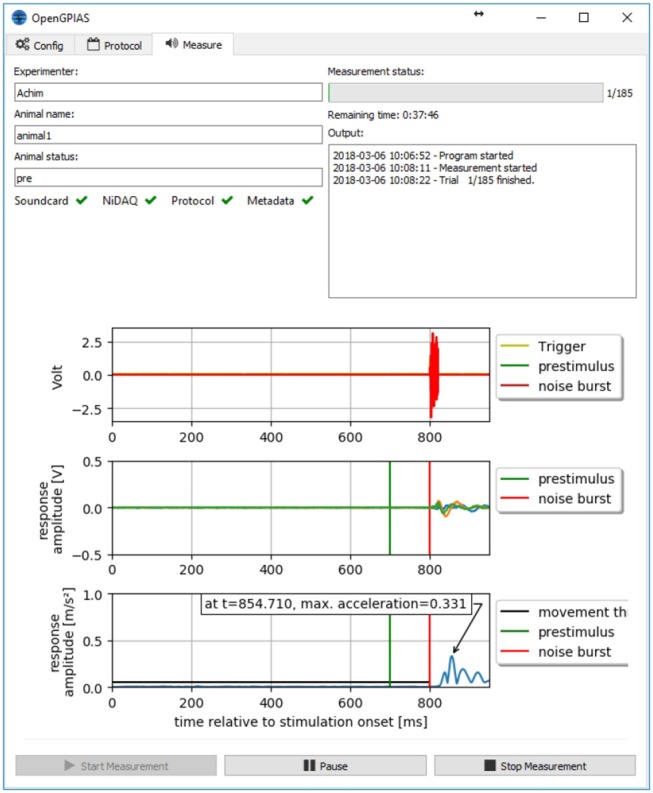
Measurement interface. The interface of the measurement program. The different tabs provide access to the different modules. The measurement module provides inputs for the meta data, a status of the components, a log of the measurement, and a plot of the data from the previous measurement.

The main part of the software is the measurement module (see Figure 4). This module checks whether both the soundcard and data acquisition card are configured properly and are ready to play or record, respectively. Furthermore, it checks the selected protocol file to be valid.

Before starting the measurement, the user has to supply some meta data, the experimenter name, the animal name, and optionally a treatment, to allow for consistent annotation and administration of the data.

When everything is ready and the animal has been placed in the restrainer, the measurement can be started. Each trial is presented to the animal using three channels of the sound card (trigger pulse, pre-stimulus, and startle stimulus) and recorded using the data acquisition card (three input channels from the acceleration sensor and three input channels directly from the soundcard). Data is saved after each trial and raw data is plotted to provide direct feedback to the experimenter.

The data is stored in a folder structure according to the meta data to allow for an organized storage. Data is saved in the form of numpy files (.npy) for efficient storage, but can be exported to ASCII files (.txt) or spreadsheet files for later evaluations.

## Results

In the following section, we show that exemplary recorded data using the novel setup is consistent with measurements from already established setups. All shown GPIAS values are median values calculated from the full combinatorial of all gap and nogap amplitudes according to Schilling et al. (2017). All statistical analysis was performed using the SciPy stats library in Python (Olifant, 2007).

**Figure 5:**
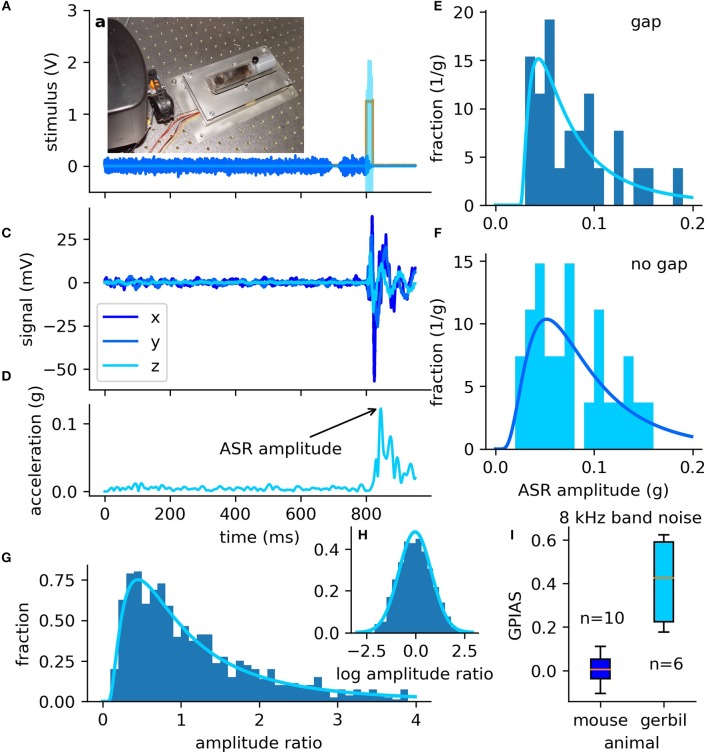
Stimuli and distribution of ASR amplitudes and GPIAS in mice. **(A–H)** Data measured in exemplary mouse. **(A)** The sensor system of the setup including a specifically designed mouse restrainer, designed to keep the mouse at a constant distance to the loudspeakers. **(B)** Output of the audio amplifier used to drive the loudspeakers (blue: band noise with gap, cyan: startle pulse, orange: trigger directly from soundcard). **(C)** Raw signal of the acceleration sensor measured with the data acquisition card for three directions. **(D)** RMS of the three channels, pre-processed with a low-pass filter (cutoff: 40 Hz). The maximum of the signal in the 150 ms interval after startle pulse onset is defined as the ASR amplitude (arrow). **(E,F)** Distributions of the ASR amplitudes measured as a response to GPIAS paradigm stimuli [without gap **(E)** and with a gap of silence **(F)**], with a center frequency of 8 kHz and a spectral width of ±1/2 octaves. Both data sets are fitted with a log-normal distribution (solid line) using the maximum likelihood estimator. **(G)** Histogram of the full combinatorial of the ratios of gap (*A*_gap_) and no gap (*A*_nogap_) ASR amplitudes. Ratio histogram (blue bars) can be described well with a log-normal distribution (solid line) as shown in a previous study (Schilling et al., 2017). **(H)** The logarithmized ratios (blue bars) are therefore Gaussian distributed (solid line). **(I)** Comparison of mice (C57/BL/6 “J”Crl) and Mongolian gerbils. Mongolian gerbils show a clear GPIAS, whereas these mice do not show any GPIAS (significant higher GPIAS of gerbils tested with two-sided Mann–Whitney-U rank sum test, *p* < 0.003).

For mouse startling, a special restrainer including a 3D printed cap was developed (cf. Figure 5A) to keep the mouse at a constant distance from the loudspeakers.

Figures 5E,F show the ASR amplitudes for one exemplary animal (mouse) stimulated according to the GPIAS paradigm (exemplary stimulus shown in Figure 5B, 30 trials, center frequency 8,000 Hz ± 1/2 octave).

The ASR amplitudes are defined as the maximum of the RMS (cf. Equation 2) of the acceleration of the 3D acceleration sensor (raw data: Figure 5C) visualized as black arrow in Figure 5D. Even the lightweight mice can induce a clearly measurable acceleration and thus a good signal-to-noise ratio.

The data is in good agreement with results from a previous study conducted with another setup (Tziridis et al., 2012; Schilling et al., 2017), showing that ASR amplitudes are not normally distributed, but the startle responses can be well described by a log-normal distribution (Figures 5E,F). Also, the ratios of gap and no gap amplitudes are log-normally distributed (Figure 5G), i.e., the logarithmic ratios are almost perfectly normally distributed (cf. Figure 5H).

The exemplary data clearly show that the here described measure for the ASR amplitudes based on the 3D acceleration vector (Equation 1) is a valid choice and leads to equivalent quantitative results as the 1D-peak-to-peak amplitudes measured with standard force sensors. The data show that the gap of silence does not lead to an inhibition of the ASR amplitude (for exemplary animal: Figure 5H, average ≈0) in our mouse strain whereas the gerbils show a clear GPIAS. Figure 5I summarizes the GPIAS values for 10 mice and 6 gerbils providing evidence that the gap leads to clear GPIAS in gerbils but not in this mouse strain (mouse vs. gerbil: *p* < 0.003).

**Figure 6:**
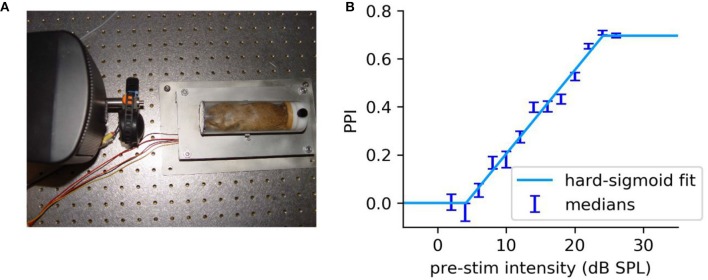
Exemplary threshold measurement of a Mongolian Gerbil. **(A)** Image of the used setup including the restrainer optimized for Mongolian Gerbils. **(B)** PPI as a function of the pre-stimulus intensity (2–26 dB SPL, 2 dB steps) for pure-tone pre-stimuli (2 kHz). Each stimulus condition was repeated 50 times. The PPI is the median of the full combinatorial PPI (cf. Schilling et al., 2017) of ASR amplitudes for pre-stimulus condition and no pre-stimulus condition. The errorbars are the standard deviation of the medians of 100,000 bootstrapped data sets and are an estimator for the standard error of medians. The stimulus-response function can be approximated with a hard-sigmoid function as described by Schilling et al. (2019), where the stimulus-response functions of neurophysiological parameters were fitted with an hard-sigmoid function. The threshold is defined as the value where the hard sigmoid function diverges form zero.

Additionally, the setup (cf. Figure 6A) can be used to estimate behavioral thresholds by presenting pure-tone pre-stimuli of varying intensity followed by a loud startle stimulus (same startle stimulus as used for GPIAS). The PPI as a function of the pre-stimulus intensity has a sigmoid shape (cf. Figure 6) and can be fitted with a hard-sigmoid function, which was already established to estimate thresholds using neurophysiological parameters (Schilling et al., 2019). For the behavioral thresholds, the lower asymptote is fixed to zero as for very low pre-stimuli the ASR reflex amplitude of pre-stimulus and no pre-stimulus condition are equal. Thus, the offset of the hard sigmoid function is zero.

## Discussion

In this study, we present an open-source setup for the measurement of acoustic startle reflexes evoked by a loud noise burst with preceding pure-tone and/or gap pre-stimuli for the estimation of hearing thresholds and identification of possible tinnitus percepts in rodents.

The pre-stimuli and startle stimuli (noise bursts) are presented using two separate loudspeakers, controlled by a commercial soundcard. The response amplitudes of the animals are captured by a 3D-acceleration sensor, whose output is digitized using a data acquisition card controlled via a Python program.

We have shown that the described setup can be used to present well-defined pure-tone and band noise stimuli and to quantify the ASR amplitudes. Additionally, we were able to show that the pre-stimuli lead to a clear GPIAS (cf. Figure 5H) in gerbils but not in the mouse strain. However, it is common knowledge that the induction of a pre-pulse inhibition in this mouse strain is very difficult (Willott et al., 2003). Additionally, we could demonstrate that the ASR amplitudes (cf. Csomor et al., 2008) as well as the ratios of gap and no gap amplitudes (cf. Schilling et al., 2017) are log-normally distributed (cf. Figures 5E,F).

Besides these findings proving the validity of the setup, it has to be mentioned that the GPIAS paradigm is discussed controversially regarding the suitability of that paradigm for tinnitus screening as several issues remain unsolved. It is not clear if the changes of the GPIAS after potential tinnitus induction by salicylate injection or noise trauma are secondary effects of e.g., hearing loss or neural plasticity (learning). Additionally, it was stated that a phantom percept with significant cortical components cannot be identified using a brainstem response paradigm (Eggermont, 2013; Eggermont and Roberts, 2015) and that the “filling in hypothesis” cannot be confirmed in human subjects (Campolo et al., 2013). For these reasons, in the last years many studies were performed to validate the GPIAS paradigm (e.g., Moreno-Paublete et al., 2017). Nevertheless, still a lot of work remains undone, thus, an open source setup can help to bring the method to many laboratories which will potentially help to address these unsolved issues.

In contrast to existing, more costly systems, our setup requires only low-cost hardware components and, with open-source software, allows for more flexible adaptation to different measurement protocols.

All our hardware components are listed in detail and the software is provided as open-source (hardware and software documentation: http://open-gpias.readthedocs.io).

## Data Availability

The datasets generated for this study are available on request to the corresponding author.

## Ethics Statement

The Mongolian gerbils were housed in standard animal racks (Bio A.S. Vent Light, Ehret Labor- und Pharmatechnik, Emmendingen, Germany) in groups of 3–4 animals with free access to water and food at a room temperature of 20–25°C under a 12/12 h dark/light circle. The mice (type: C57/BL/6 “J”Crl) were housed in groups of two animals under equal conditions. The care of the animals was approved by the state of Bavaria (Regierungspräsidium Mittelfranken, Ansbach, Germany, No. 54-2532.1-02/13 and Regierungspräsidium, Würzburg, Germany, No. 55.2-2532-2-137). Exemplary measurements were recorded using 8 male Mongolian gerbils purchased from the Leibniz Institute for Neurobiology in Magdeburg and 10 male mice purchased from Charles River Laboratories.

## Author Contributions

AS designed the research. AS, RG, HR, MS, and JG performed the research. AS, RG, HR, and MS analyzed the data and developed the setup software and hardware. AS, RG, PK, CM, KT, MG, HS, and WK wrote the paper.

### Conflict of Interest Statement

The authors declare that the research was conducted in the absence of any commercial or financial relationships that could be construed as a potential conflict of interest.
